# Aquaporin 4-specific T cells and NMO-IgG cause primary retinal damage in experimental NMO/SD

**DOI:** 10.1186/s40478-016-0355-y

**Published:** 2016-08-08

**Authors:** Bleranda Zeka, Maria Hastermann, Nathalie Kaufmann, Kathrin Schanda, Marko Pende, Tatsuro Misu, Paulus Rommer, Kazuo Fujihara, Ichiro Nakashima, Charlotte Dahle, Fritz Leutmezer, Markus Reindl, Hans Lassmann, Monika Bradl

**Affiliations:** 1 Department of Neuroimmunology, Center for Brain Research, Medical University Vienna, Spitalgasse 4, A-1090 Vienna, Austria; 2 Clinical Department of Neurology, Innsbruck Medical University, Innsbruck, Austria; 3 Medical University Vienna, Section for Bioelectronics, Center for Brain Research, Vienna, Austria; 4 Department of Neurology, Tohoku University Graduate School of Medicine, Sendai, Japan; 5 Department of Neurology, Medical University Vienna, Wien, Austria; 6 Department of Clinical and Experimental Medicine, Faculty of Health Sciences, Linköping University, Linköping, Sweden

## Abstract

Neuromyelitis optica/spectrum disorder (NMO/SD) is a severe, inflammatory disease of the central nervous system (CNS). In the majority of patients, it is associated with the presence of pathogenic serum autoantibodies (the so-called NMO-IgGs) directed against the water channel aquaporin 4 (AQP4), and with the formation of large, astrocyte-destructive lesions in spinal cord and optic nerves. A large number of recent studies using optical coherence tomography (OCT) demonstrated that damage to optic nerves in NMO/SD is also associated with retinal injury, as evidenced by retinal nerve fiber layer (RNFL) thinning and microcystic inner nuclear layer abnormalities. These studies concluded that retinal injury in NMO/SD patients results from secondary neurodegeneration triggered by optic neuritis.

However, the eye also contains cells expressing AQP4, i.e., Müller cells and astrocytes in the retina, epithelial cells of the ciliary body, and epithelial cells of the iris, which raised the question whether the eye can also be a primary target in NMO/SD. Here, we addressed this point in experimental NMO/SD (ENMO) induced in Lewis rat by transfer of AQP4_268–285_-specific T cells and NMO-IgG.

We show that these animals show retinitis and subsequent dysfunction/damage of retinal axons and neurons, and that this pathology occurs independently of the action of NMO-IgG. We further show that in the retinae of ENMO animals Müller cell side branches lose AQP4 reactivity, while retinal astrocytes and Müller cell processes in the RNFL/ganglionic cell layers are spared. These changes only occur in the presence of both AQP4_268–285_-specific T cells and NMO-IgG.

Cumulatively, our data show that damage to retinal cells can be a primary event in NMO/SD.

## Introduction

Optic nerves and spinal cord are preferential targets of inflammation in NMO/SD, an astrocytopathic disease of the central nervous system (CNS) associated with the presence of pathogenic serum autoantibodies directed against AQP4 [1–3]. A large number of recent studies using optical coherence tomography (OCT) demonstrated that damage to optic nerves in NMO/SD is also associated with retinal injury [4]. This finding raised the questions whether retinal injury in NMO/SD patients only results from secondary neurodegeneration triggered by optic neuritis, whether it may also be a consequence of retinal inflammation initiated by AQP4-specific T cells, and whether there is a contribution of pathogenic AQP4-specific antibodies to this process. These questions were especially important since AQP4, the target antigen for both, is expressed in the eye: by Müller cells and astrocytes in the retina [5], and by epithelial cells of the ciliary body and the iris [6]. To address these points, we searched for ocular inflammation in experimental NMO/SD (ENMO).

## Materials and methods

### Animals

All animal experiments were approved by the Ethic Commission of the Medical University Vienna and performed with the license of the Austrian Ministery for Science and Research (GZ66.009/195-WF-V-3b/2015;GZ66.009/0241-WF/II/3b/2014). Lewis rats were obtained from Charles River Wiga (Sulzfeld, Germany), and were used at an age of 7–8 weeks. During the experiments, they were housed in the Decentral Facilities of the Institute for Biomedical Research (Medical University Vienna) under standardized conditions.

### T cells and immunoglobulins used in transfer experiments

The T cells used were specific for rat AQP4_268–285_ (KAAQQTKGSYMEVEDNRS) which contains two overlapping epitopes for antigen presentation via RT1.B^L^: QQTKGSYME, and TKGSYMEVE, and were grown under culture conditions selecting the T-helper 1 subset of CD4^+^ T cells [7–9].

The plasmapheresates used as sources for NMO-IgG were termed NMO-IgG_9_, NMO-IgG_V_, and NMO-IgG_S_. NMO-IgG_9_ derived from a Japanese NMO/SD patient with optic neuritis only, NMO-IgG_V_ from an Austrian NMO/SD patient with optic neuritis followed 5 months later by myelitis, and NMO-IgG_S_ from a Swedish NMO/SD patient with repeated optic neuritis and myelitis, and with additional MS-typical brain lesions. NMO-IgG_9_ and NMO-IgG_V_ were purified using Protein G Sepharose 4 Fast Flow (GE Healthcare Bio-Sciences, Pasching, Austria) according to the manufacturer’s instructions, and adjusted to a concentration of 10 mg/ml. NMO-IgG_S_ was injected as plasmapheresate without further purification. The use of the plasmapherisates/NMO-IgG preparations for research was approved by the Ethics Committee of Tohoku University School of Medicine (No. 2007–327), by the Regional and National Ethical Committee of Sweden (2013/153-31 Linköping), and by the Ethics Committees of the Medical University of Vienna (No. 1005/2014).

As negative control (co-IgG), commercially available normal human IgG (Subcuvia™, Baxter, Vienna) was used, diluted with phosphate buffered saline (PBS) to an IgG concentration of 10 mg/ml prior to use.

### Induction of ENMO and tissue preparation

ENMO was induced by intraperitoneal injection of 1x10^7^ AQP4_268–285_-specific T cells on day 0, followed by intraperitoneal injection of NMO-IgG on day 4 or 5. A few animals received 3x10^6^ AQP4_268–285_-specific T cells on day 0, followed by intraperitoneal injection of NMO-IgG on day 5. The animals were then killed 24–48 h later with CO_2_ and perfused with 4 % paraformaldehyde in phosphate-buffered saline. The eyes were dissected, immersed for another 18–24 h in PFA and embedded in paraffin for histological analysis.

### Histological and immunohistochemical analysis

All stainings were done essentially as described [10, 11] using rabbit polyclonal antibodies against AQP4 (Sigma, Germany), rabbit polyclonal against glial fibrillary acidic protein (GFAP; from Dako, Denmark), rabbit polyclonal antibodies against CD3 (to stain T cells; NeoMarkers, Fremont, USA), rabbit polyclonal antibodies against nitric oxide synthetase II (AB16311; Merck Millipore, Darmstadt, Germany), the mouse monoclonal antibody ED1 (to stain macrophages and activated microglia; Serotec, Germany), the mouse monoclonal anti-glutamine synthetase antibody (BD Biosciences, San Jose, USA), anti-human immunoglobulin (biotinylated donkey; polyclonal; Amersham, UK), anti-complement C9 (rabbit polyclonal [12]), and mouse monoclonal antibodies against amyloid-beta precursor protein (APP; Merck Millipore, Massachusetts, USA).

### Counting and statistics

Counting was performed manually at 100 ×, 200 × or 400 × magnification using a morphometric grid. One slide contained two sagittal sections of one eye and one coronal section of the contralateral eye of the same animal. For quantification of the number of T cells in the retina, ciliary body, and iris, the largest inflammatory lesion per slide was chosen for each animal studied, and counted at 100 × magnification. For quantification of the number of T cells in different layers of the retina, one representative inflammatory lesion per slide and animal was chosen and counted at 100 × magnification. For quantification of the number of T cells proximal or distal to the optic nerve T cells in the whole proximal or distal sagittal sections were counted. To determine the number of APP^+^ spheroids, the largest lesion with APP^+^ structures was counted in the retina, papilla, and in the center and periphery of the optic nerve, using a 400 × magnification.

Statistics were calculated with IBM SPSS Statistics 21. The Mann-Whitney (Wilcoxon) U test and student’s t-test were used for statistical analysis. For multiple comparison corrections Bonferroni and Bonferroni-Holm was used.

## Results

### The anterior visual pathway in ENMO

**Fig. 1:**
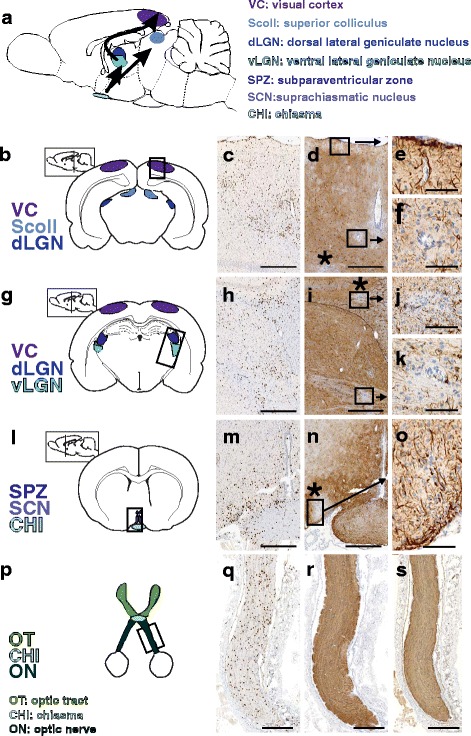
Inflammation and astrocyte-destructive lesions in the anterior visual pathway. Shown here is a scheme of the anterior visual pathway in rats (**a**) and schemes of cross sections through the brain at different levels along the neuraxis to show important areas of the anterior visual pathway (**b**,**g**,**l**,**p**) which were then analyzed in ENMO rats following staining with anti-CD3 antibodies (*brown*) to visualize T cell infiltrates (**c**,**h**,**m**,**q**), and with anti-AQP4 antibodies (*brown*) to visualize loss of AQP4 reactivity (**d**,**i**,**n**,**r**). *: lesions with AQP4 loss outside the anterior visual pathway. *Black* boxes: selected regions shown after staining with anti-GFAP antibodies (*brown*, **e**,**f**,**j**,**k**,**o**,**s**). Hematoxylin staining was used to reveal nuclei (*blue*). For all drawings, schemes provided by Paxinos and Watson [38] and by Mueller [39] served as guide lines. Bars = 500 μm (**c**,**d**,**h**,**i**,**m**,**n**), 300 μm (**q**,**r**,**s**), and 100 μm (**e**,**f**,**j**,**k**,**o**)

In ENMO, brain lesions with AQP4 loss occur on a background of diffuse T cell infiltration, are found in many different sites and may hit structures of the anterior visual pathway, but do not specifically target them (Fig. 1). Also the optic nerves of ENMO rats contain many T cells, but astrocyte-destructive lesions are essentially absent (Fig. 1).

### Intraocular T cells in ENMO

**Fig. 2:**
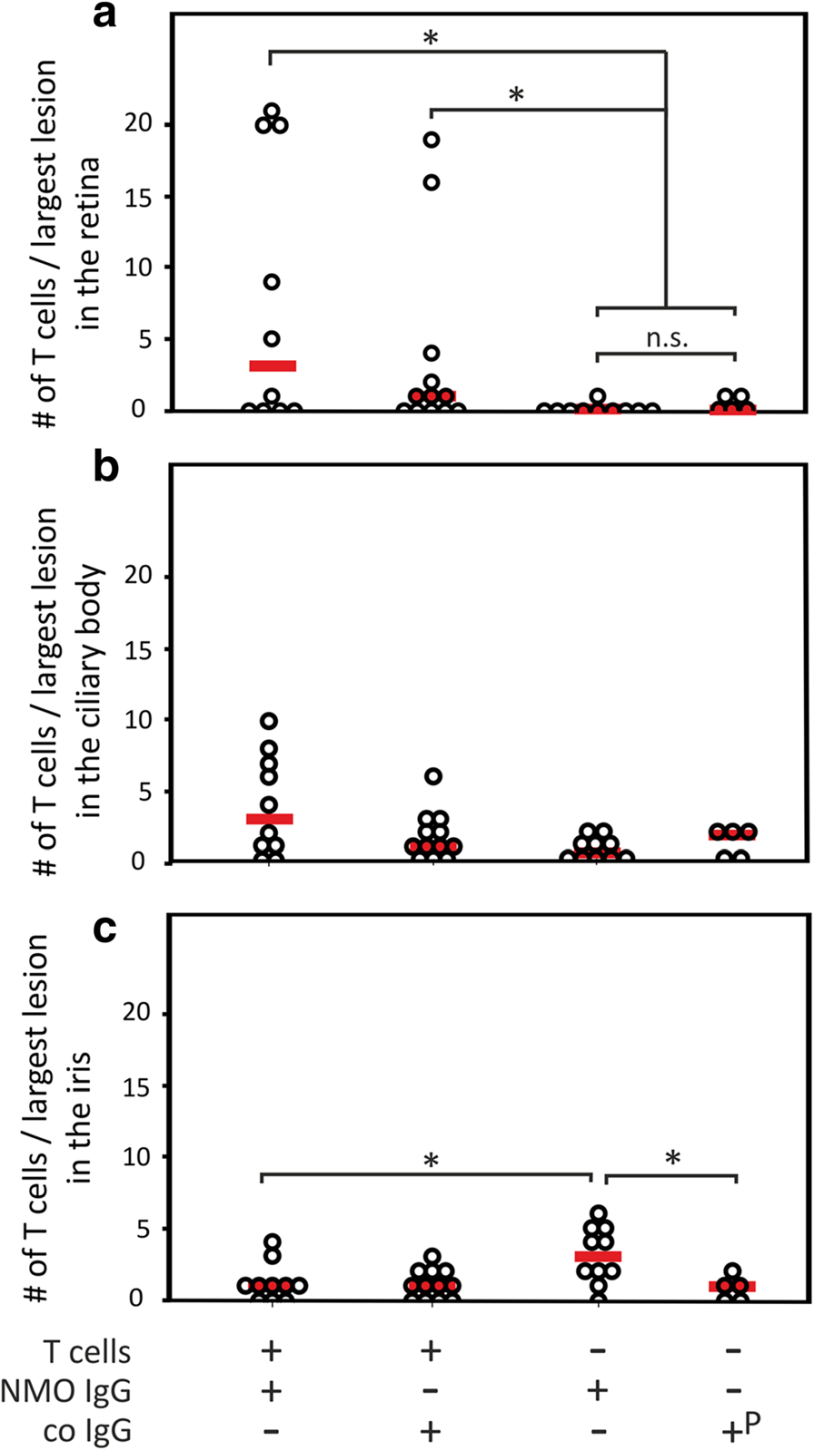
Retinitis in the eye of animals with ENMO. Median T cell numbers seen in the largest lesion within the retina (**a**), ciliary body (**b**) and iris (**c**) per rat after injection of AQP4_268–285_-specific T cells and NMO-IgG (*n* = 10), AQP4_268–285_-specific T cells and control IgG (*n* = 12), NMO-IgG (*n* = 10), and control IgG (*n* = 3) or PBS (*n* = 2). The animals receiving control IgG and PBS were pooled (^P^). (a) In the retina, differences between NMO-IgG injected and control IgG or PBS injected rats were not significant (*p* = 0.505), while differences between this group and the AQP4_268–285_-specific T cells and NMO-IgG injected and the AQP4_268–285_-specific T cells and control IgG injected groups were significant (* *p* = 0.03 and 0.044, respectively). (b) In the ciliary body, there were no statistically significant differences between the 4 different groups after correction for multiple comparison. (c) In the iris, statistically higher numbers of T cells were found in animals injected with NMO-IgG only in comparison to the AQP4_268–285_-specific T cells and NMO-IgG and the co IgG/PBS injected groups (* *p* = 0.038 and 0.031, respectively,). In all cases, the Mann-Whitney exact U test with Bonferroni correction for multiple comparisons was used

Transfer of AQP4_268–285_-specific T cells (with or without NMO-IgG) into Lewis rats led to T cell infiltrates in retina and ciliary body. However, only the retinal T cell infiltration remained statistically significant, when these rats were compared to their counterparts injected with antibodies only (Fig. 2). In the iris, there was no increase in T cell numbers after injection of AQP4_268–285_-specific T cells and NMO-IgG. In fact, T cell numbers were even statistically higher in animals injected with NMO-IgG only, and in NMO-IgG injected rats compared to the co IgG/PBS group. However, since the overall numbers of T cells at this site were very low, we did not further follow up this point.

**Fig. 3:**
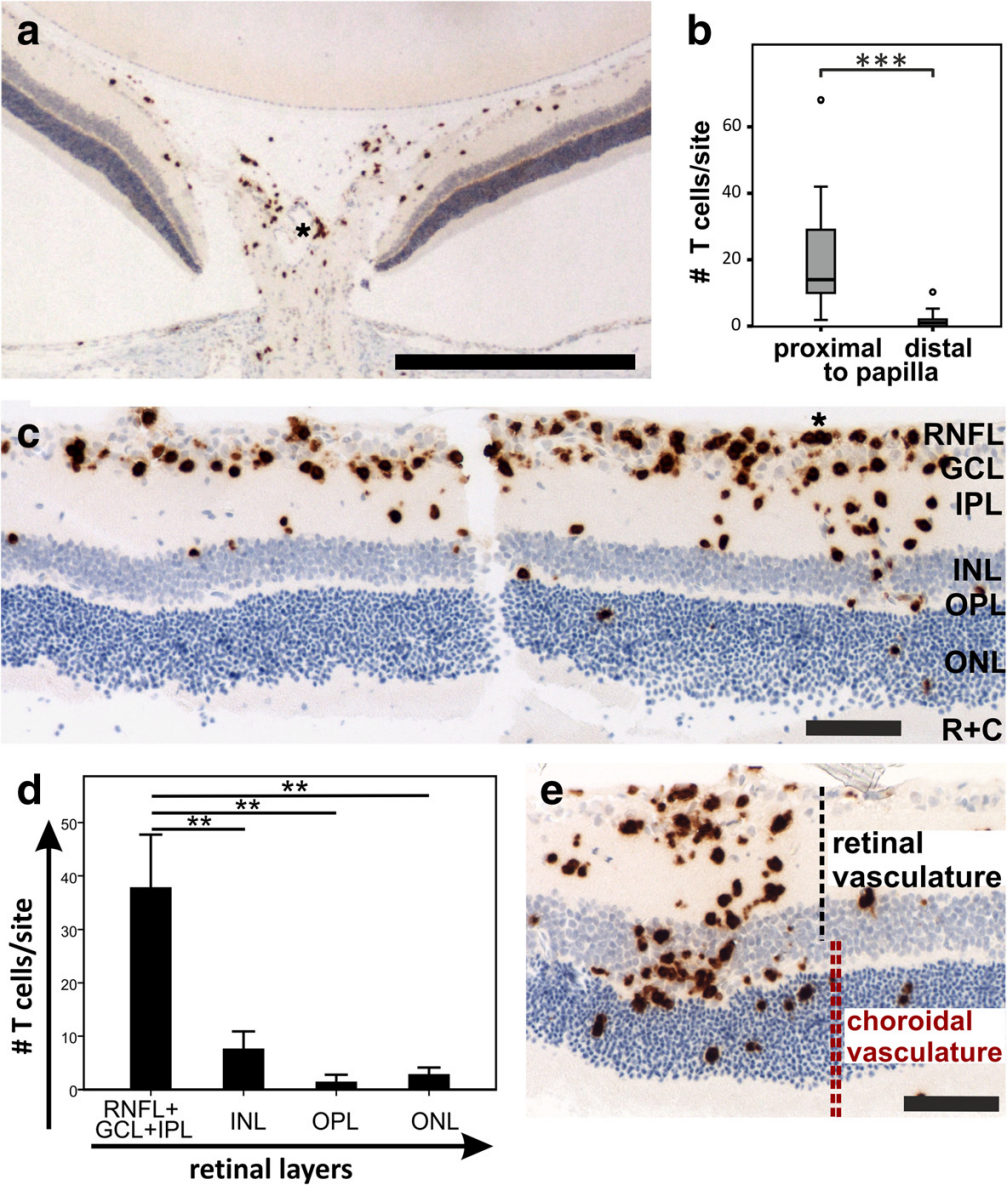
Two gradients of T cell infiltration into the retina. **a** CD3^+^ T cells (*brown*) in papilla and peripapillary retina. **b** statistically significant differences in T cell numbers proximal and distal from the papilla, as determined from 1 whole retinal section each of 9 and 13 rats, respectively (*p* = 0.000076 according to Mann-Whitney U test; plots show the range of T cell numbers (whiskers) with 50 % scores (interquartile range, boxes) centered around median values (horizontal lines within boxes), and mild outliers (circles). **c** Retinal cross section show an inside - > outside gradient of CD3^+^ T cells (*brown*). RNFL: retinal nerve fiber layer, GCL: ganglionic cell layer, IPL: inner plexiform layer, INL: inner nuclear layer, OPL: outer plexiform layer, ONL: outer nuclear layer, R + C: layer of rods and cones. **d** Quantification of T cell infiltration in different retinal layers, as determined from one representative lesion/rat (*n* = 5 rats; **p = 0.003, t-test, Bonferroni-correction for multiple comparisons). **e** Invasion of CD3^+^ T cells (*brown*) from vessels spanning the different retinal layers. The contribution of retinal and choroid vasculature to the blood supply is indicated. Bars = 525 μm (**a**) and 100 μm (**c**,**e**). All histological sections were counterstained with hematoxylin to show nuclei in blue; (*) perivascular T cell clusters

Instead, we next only studied the retina in more detail. Since in these initial ocular studies, 3x10^6^ AQP4_268–285_-specific T cells have been injected, which was not sufficient to induce retinitis in all experimental animals (Fig. 2), we used for all following experiments 1x10^7^ AQP4_268–285_-specific T cells and NMO-IgG to induce ENMO, to have a more stable model for studying the consequences of T cell infiltration into the retina. We found, that retinal T cell distribution followed two different gradients. The first gradient was proximal - > distal of the papilla, with most retinal T cells and perivascular T cell clusters found in close vicinity to or within the optic nerve head (Fig. 3). The second gradient was an inside - > outside gradient of infiltration, in which the highest numbers of intraparenchymal and perivascular retinal T cells was seen in the nerve fiber/ganglion cell/inner plexiform layers, lower numbers in the outer plexiform and nuclear layers, and essentially no T cells in the layers of rods and cones (Fig. 3). Sometimes, this gradient was interrupted by rare areas of perivascular T cell infiltration at vessels crossing through several retinal layers, mostly involving the outer nuclear/inner plexiform/inner nuclear layers (Fig. 3). Rarely, we also saw T cells in retinal pigment epithelium and choroid, but did not follow this up further (data not shown).

### In ENMO, AQP4-specific T cells cause axonal pathology predominantly in the retinal nerve fiber/ganglionic cell layer

**Fig. 4:**
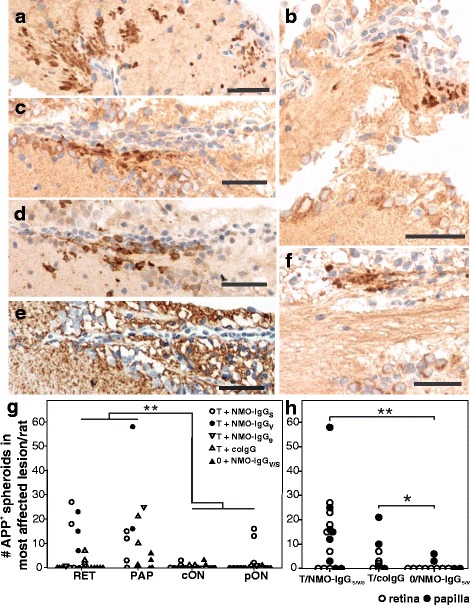
Axonal spheroids/end bulbs in retina, papilla, and optic nerve. Sections through the optic nerve head (**a**, **b**), ganglionic cell layer (**c**-**e**, consecutive sections) and retinal nerve fiber layer (**f**) were reacted with antibodies against APP (**a**-**c**, **f**), iNOS (**d**), and AQP4 (**e**). Antibody binding is shown in *brown*, and the nuclei are revealed by counterstaining with hematoxylin. The tissue sections derived from Lewis rats injected with AQP4_268–285_-specific T cells and either NMO-IgG (**a**,**c**-**f**) or co-IgG (**b**). APP^+^ axonal spheroids/endbulbs can be seen upon the induction of inflammation by AQP4_268–285_-specific T cells in presence and absence of NMO-IgG (**a**-**c**, **f**), and coincide with iNOS^+^ macrophages (**d**) and essentially normal AQP4 expression (**e**). Bars = 25 μm. **g** Numbers of APP^+^ spheroids/endbulbs in the most affected lesion per rat, found in retina (RET), papilla (PAP), and central or peripheral parts of optic nerve cross sections (cON or pON, respectively), as determined from 11 rats injected with T cells and NMO-IgGs, and from 8 rats injected with NMO-IgGs only. The numbers of APP^+^ spheroids/end bulbs were significantly higher in retina and papilla compared to central and peripheral optic nerve (*p* = 0,002, Mann-Whitney U-test). **h** Numbers of APP^+^ spheroids/endbulbs in the most affected retinal and papillary lesion per rat, found in animals injected with AQP4_268–285_-specific T cells/NMO-IgG (*n* = 15; NMO-IgG from 3 different sources), AQP4_268–285_-specific T cells/control IgG (coIgG) (*n* = 8) and NMO-IgG only (*n* = 12). The differences between the T cell injected groups and the group receiving NMO-IgG only were significant (**p = 0.006 and *p = 0.025, respectively; Mann-Whitney U-test with Bonferroni-Holm correction)

In NMO/SD patients, thinning of the retinal nerve fiber and ganglion cell layers is considered a secondary consequence of optic neuritis [4]. However, in EAE models, encephalitogenic T cells are able to directly cause transient and permanent injury of neurons, as revealed by the formation of axonal spheroids and end bulbs [13]. Is there evidence for axonal injury in the retina of our ENMO animals? To address this question, we next searched for APP^+^ spheroids/end bulbs and iNOS^+^ myeloid cells at this site. We found inflammatory lesions with APP^+^ axonal spheroids/end bulbs in the retinal nerve fiber/ganglionic cell layer, and significantly more APP^+^ structures in retina and papilla than in the extra-ocular optic nerve. These pathological changes could be clearly ascribed to the action of AQP4_268–285_-specific T cells, since T cells triggered the formation of APP^+^ structures both in the presence and in the absence of NMO-IgG (Fig. 4). Retinal APP^+^ axonal spheroids/end bulbs co-localized with iNOS^+^ macrophages/microglia (Fig. 4).

### In ENMO, RNFL astrocytes are spared and Müller cells are damaged

The healthy retina is protected from serum antibodies by the inner and outer blood–retinal barriers (BRB) (30), but these barriers can be opened by inflammatory T cells, allowing the entry of NMO-IgG. Could this cause antibody-mediated damage to AQP4-expressing retinal astrocytes or Müller cells?

**Fig. 5:**
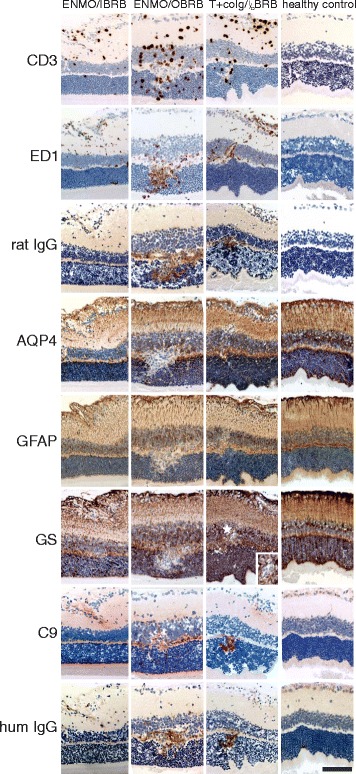
Histological characterization of inflammatory lesions in the retina. Consecutive retinal sections were reacted with antibodies against CD3, ED1, rat IgG, AQP4, GFAP, glutamine synthetase (GS), C9, and human IgG. Positive reaction products are shown in brown or red (C9 only). All sections were counterstained with hematoxylin to reveal nuclei in blue. Bars = 100 μm. The tissue derived from animals injected with AQP4_268–285_-specific T cells and NMO-IgG (ENMO), with AQP4_268–285_-specific T cells and human control IgG (T + co IgG) and healthy controls, and show inflammation at the inner blood retinal barrier (IBRB), at the outer blood retinal barrier (OBRB), simultaneously at both retinal barriers (^I^/_O_BRB), or an absence of inflammation at these sites in the control animals. The insert shown for the GS stained retina of the rat injected with T cells and co IgG refers to the area labeled by a white star in the adjacent overview. It demonstrates that there is no loss of GS reactivity at this site, but the lumen of a blood vessel. Bar = 100 μm

To address this question, we first concentrated on the retinal nerve fiber/ganglionic cell layer containing most retinal astrocytes [14] and the terminal processes of Müller cells [15]. We did not see any evidence for AQP4 loss or for the formation of astrocyte-destructive lesions in these layers (Table 1): There was only little leakage of serum proteins like rat and human IgG and complement at the retinal nerve fiber/ganglionic cell layer, and we did not see the accentuated surface staining of astrocytic or Müller cell processes indicative of IgG or complement deposition on these cells (Fig. 5).

**Table 1:** The numbers of perivascular cuffs (total numbers/numbers with AQP4 loss) in retinal nerve fiber layer (RNFL), inner nuclear layer (INL), outer plexiform layer (OPL), and outer nuclear layer (ONL), as determined from one retinal section/rat

Treatment	# rats	# of perivascular cuffs in RNFL (total/AQP4 loss)	# of perivascular cuffs in INL/OPL/ONL (total/with AQP4 loss)
T + NMO-IgG_S_	5	5/0	3/1
T + NMO-IgG_V_	4	8/0	12/8
T + NMO-IgG_9_	2	6/0	n.d.
T + co-IgG	4	17/0	8/0

Please note that not every inflammatory lesion in the INL/OPL/ONL region was associated with AQP4 loss. This was also observed in spinal cord sections of Lewis rats with experimental NMO [9, 11]

**Fig. 6:**
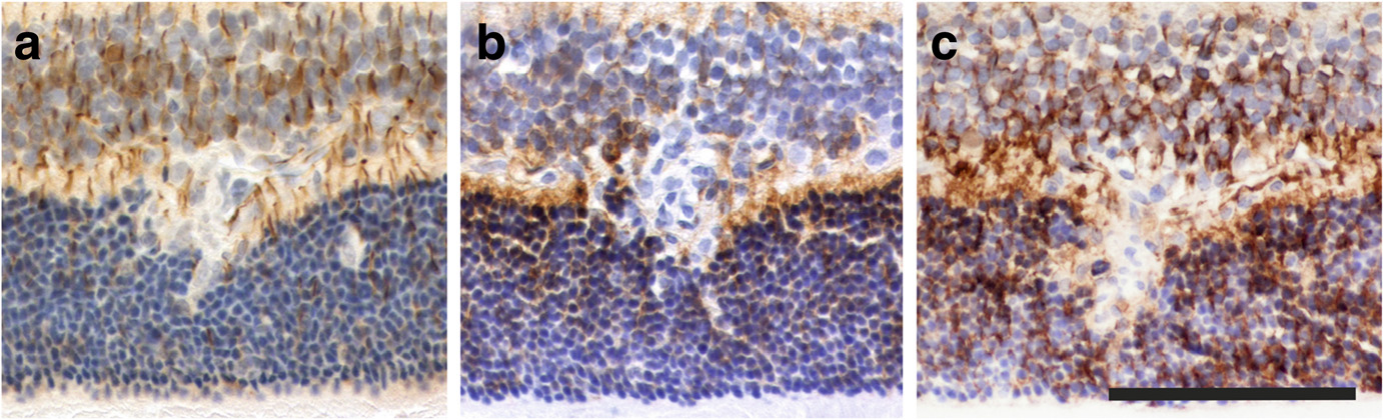
Histological characterization of AQP4 loss in the outer plexiform layer. Consecutive sections of an inflammatory retinal lesion of an animal injected with AQP4_268–285_-specific T cells and NMO-IgG, reacted with antibodies against GFAP (**a**), AQP4 (**b**) and GS (**c**). Bar = 100 μm

Next, we concentrated on inflammatory lesions in the inner plexiform/inner nuclear/outer plexiform/outer nuclear layers, where leakage of complement proteins and of rat and human IgG was more severe than the leakage seen in the retinal nerve fiber/ganglionic cell layer (Fig. 5), and where NMO-IgG should primarily target Müller cells, which have their perikarya in the inner nuclear layer, their stem processes spanning throughout the entire thickness of the retina, and their side branches in the outer and inner plexiform layers [14, 15] (Fig. 5). We found lesions with AQP4 loss in the outer plexiform layer (Table 1). To make sure that the AQP4 loss in the INL/OPL/ONL area was not a pathological feature unique to one NMO-IgG only, we also tested NMO-IgG from different sources (NMO-IgG_s_, NMO-IgG_V_, or NMO-IgG_9_). We observed AQP4 loss with NMO-IgG preparations from different patients (Table 1). The loss of AQP4 reactivity in the OPL was not caused by a cellular displacement due to inflammatory infiltrates, since there was no accumulation of glutamine synthetase or of GFAP expressing cells/cellular processes in the perilesional area (Figs. 5 and 6). We also did not see any evidence for deposition of C9 or human IgG on the surface of Müller cell bodies or stem processes (Figs. 5 and 6). AQP4 loss was only seen in the presence of both AQP4_268–285_-specific T cells and NMO-IgG.

## Discussion

We show here that damage to retinal axons and neurons in ENMO can be a primary event provoked by the induction of inflammatory lesions. These pathological changes could be clearly ascribed to the action of AQP4_268–285_-specific T cells, since T cells triggered the formation of APP^+^ structures both in the presence and in the absence of NMO-IgG. We further show that in ENMO, Müller cell side branches in the outer plexiform layer lose AQP4 reactivity. This effect is only seen when AQP4_268–285_-specific T cells induced inflammatory lesions in the presence of NMO-IgG.

The induction of ocular inflammation is not a unique feature of AQP4_268–285_-specific T cells, but also occurs when sufficient numbers of activated T cells specific for other ocular antigens are found in the circulation. Examples are T cells specific for the astrocytic S100β protein [16], for myelin basic protein (MBP) [17–19] which is a component of myelinated nerve bundles in the iris [20], and for the retinal soluble antigen (S-Ag) [21] or the cellular retinaldehyde binding protein (CRALBP) [22].

In the inflamed retina, AQP4_268–285_-specific T cells are predominantly found both within the parenchyma and within perivascular cuffs in the optic nerve head and in the peripapillary retina, and display two gradients of cellular distribution: the first one proximal - > distal of the optic nerve head, and the second one from inner - > outer retinal layers. The proximal - > distal gradient can be explained by anatomical properties of the vasculature: T cells enter at postcapillary venules, capillaries from the surface of the papilla are in direct continuation with capillaries of the peripapillary retina [23], and the number of capillary layers decreases from four in the peripapillary inner retina to two and one in more centrifugally located inner retinal parts [24]. The gradient from inner - > outer retinal layers reveals that the majority of T cells infiltrating the retina comes from the retinal vasculature which forms the inner blood-retina barrier [25]. Interspersed into these gradients is a small population of T cells in close vicinity to blood vessels spanning through the different retinal layers. At least some of these cells might cross through the choroidal vasculature which comprises the outer blood-retina barrier [25, 26].

Retinal T cell infiltration causes axonal pathology in the RNFL and in the optic nerve head, as evidenced by the presence of APP^+^ axonal spheroids/end bulbs. In the RNFL, these structures were associated with iNOS^+^ activated microglia/macrophages, in line with a breakdown of the inner blood-retinal barrier by pro-inflammatory cytokines [27] and reactive oxygen and nitrogen species [28]. Possibly, the unmyelinated phenotype of retinal axons renders them especially susceptible to the damaging action of reactive nitric oxide species. The numbers of lesions with axonal pathology per retinal section were too small (on average 0.3/retinal section) to warrant translation of axonal dysfunction/loss into a measurable thinning of the RNFL. In addition, the absence of a macula in rats [29] also precludes a recapitulation of the macular thinning observed in NMO/SD patients. In the papilla, APP^+^ axonal spheroids/end bulbs were not associated with iNOS^+^ microglia/macrophages. This could suggest either that axonal pathology was initiated more proximally to the neuronal cell body, i.e., in the retinal nerve fiber layer/ganglionic cell layer, or different ages of inflammatory lesions in papilla and retinal nerve fiber/ganglionic cell layer, since iNOS expression by microglia/macrophages is temporally restricted [13]. We did not find APP^+^ axonal spheroids/end bulbs in the outer plexiform/inner nuclear layer, although there were lesions with iNOS^+^ cells at this site. On the one hand, this could be due to immunomodulatory mediators acting at the outer blood-retinal barrier, among them interleukin-10 and pigment epithelium-derived factor, which could skew myeloid cells towards higher phagocytic actions [25, 30]. On the other hand, the axons of horizontal/bipolar cells found at this site might be too short to form spheroids/end bulbs detectable by our staining technique. Hence, axonal dysfunction/loss in the RNFL is a primary outcome of ENMO, but not the only one.

Müller cell side branches in the outer plexiform layer lose AQP4 reactivity, while the terminal processes of Müller cells and astrocytes in the RNFL remain intact. The most likely explanation for this finding is the higher amount of leakage of IgG and complement at the outer blood-retina barrier than at the inner blood-retina barrier. The AQP4 loss of Müller cell side branches was not a consequence of complement-dependent (CDC) or of antibody dependent cellular cytotoxicity (ADCC), since there was no evidence for complement or immunoglobulin deposition on these cells. In this aspect, our observations in ENMO animals reflect recent findings in 3 retinae from NMO/SD autopsy cases, where Müller cells displayed a unique dynamics with scattered loss of AQP4 reactivity, and where the authors also concluded that the pathological process in the retina may not involve CDC or ADCC [31]. We do not know yet whether the scattered loss of AQP4 reactivity on Müller cells is caused by internalization of antibody-loaded AQP4, or whether it reflects the response of these cells to inflammatory cytokines or to injury of retinal neurons. The local loss of AQP4 reactivity on Müller cell processes in the outer plexiform layer could render these cells more susceptible to osmotic stress without changing their expression levels of GFAP, in line with previous findings in AQP4 null mice [32, 33]. We also do not know yet how long the effects on Müller cells last – especially since we saw loss of AQP4 reactivity under conditions of T cell infiltration and microglia activation/macrophage recruitment, while the 3 human retinae described did not contain T cells or B cells [31]. There is currently still some controversy about the role of T cells in NMO/SD. However, since T cells are found in early active lesions of NMO/SD patients [8, 34], and since AQP4-specific T cells are found in the normal immune repertoire of humans, and are expanded in the repertoire of NMO/SD patients [35–37], their possible contribution to retinal pathology in NMO/SD patients should not be ignored. Further studies are needed to specifically address this point.

Cumulatively, however, our pathological findings in the ENMO retina, and the recent study on NMO/SD retinae [31] come both to the same conclusion: Damage to the retina in NMO/SD may not only be caused by secondary retrograde changes after optic neuritis, but may also be a primary outcome of the disease.

## Conclusions

We showed here that rats with ENMO developed retinitis, which was associated with two types of retinal damage: With the formation of APP^+^ axonal spheroids/end bulbs in retina and papilla which could be clearly ascribed to the action of AQP4_268-285_-specific T cells, and with the loss of AQP4 reactivity in Müller cell side branches in the outer plexiform layer which was only seen when AQP4_268-285_-specific T cells and NMO-IgG were present. Currently, it remains unclear whether such pathological changes also occur in the retina of NMO/SD patients. To address this issue, peripapillary retinae of these patients have to be studied at the peak of optic neuritis.
